# Normative NeuroFlexor data for detection of spasticity after stroke: a cross-sectional study

**DOI:** 10.1186/s12984-016-0133-x

**Published:** 2016-03-18

**Authors:** Gaia Valentina Pennati, Jeanette Plantin, Jörgen Borg, Påvel G Lindberg

**Affiliations:** Division of Rehabilitation Medicine, Department of Clinical Sciences Karolinska Institutet, Danderyd University Hospital, Stockholm, SE-182 88 Sweden; FR3636 CNRS, Université Paris Descartes, Sorbonne Paris Cité, 75006 Paris, France; Centre de Psychiatrie et Neurosciences, Inserm U894, 75014 Paris, France

**Keywords:** Stroke, Muscle spasticity, Upper extremity, Biomechanics, Normative data

## Abstract

**Background and Objective:**

The NeuroFlexor is a novel instrument for quantification of neural, viscous and elastic components of passive movement resistance. The aim of this study was to provide normative data and cut-off values from healthy subjects and to use these to explore signs of spasticity at the wrist and fingers in patients recovering from stroke.

**Methods:**

107 healthy subjects (age range 28–68 years; 51 % females) and 39 stroke patients (age range 33–69 years; 33 % females), 2–4 weeks after stroke, were assessed with the NeuroFlexor. Cut-off values based on mean + 3SD of the reference data were calculated. In patients, the modified Ashworth scale (MAS) was also applied.

**Results:**

In healthy subjects, neural component was 0.8 ± 0.9 N (mean ± SD), elastic component was 2.7 ± 1.1 N, viscous component was 0.3 ± 0.3 N and resting tension was 5.9 ± 1 N. Age only correlated with elastic component (*r* = −0.3, *p* = 0.01). Elasticity and resting tension were higher in males compared to females (*p* = 0.001) and both correlated positively with height (*p* = 0.01). Values above healthy population cut-off were observed in 16 patients (41 %) for neural component, in 2 (5 %) for elastic component and in 23 (59 %) for viscous component. Neural component above cut-off did not correspond well to MAS ratings. Ten patients with MAS = 0 had neural component values above cut-off and five patients with MAS ≥ 1 had neural component within normal range.

**Conclusion:**

This study provides NeuroFlexor cut-off values that are useful for detection of spasticity in the early phase after stroke.

## Background

Spasticity is one of the positive signs of the upper motor neuron syndrome (UMNS) and is commonly defined according to Lance as “a motor disorder characterized by a velocity-dependent increase in tonic stretch reflexes with exaggerated tendon jerks, resulting from hyperexcitability of the stretch reflex” [1]. Spasticity occurs in a number of neurological conditions and may contribute to impaired body functions and activity limitations after stroke [2–4]. The prevalence of spasticity is 20–25 % after first-ever stroke, as assessed with conventional clinical evaluation methods such as the modified Ashworth scale [3–7]. Spasticity development is highly variable between individuals even though poor sensorimotor function is an identified risk factor [8–10].

The most commonly used clinical scale of spasticity is the modified Ashworth Scale (MAS) [11]. However, the MAS is a subjective scale with limited validity and reliability [12–14]. Another limitation is that the MAS does not allow separate measurement of neural (reflex) and non-neural (muscle and connective tissue) contributions to resistance to passive stretch. Other clinical methods, such as the REPAS [15] and the Tardieu scale [16] may enhance the diagnostic accuracy but do not objectively quantify spasticity. Thus, there is a generally recognized need for new and easy to use methods that enable more accurate and reliable evaluation of spasticity and which can help optimize choice and timing of treatments [17]. Spasticity may be managed by a wide variety of therapeutic interventions including physical therapy, pharmacological agents and surgical treatment [4]. There is now consistent evidence that spasticity after stroke can be significantly reduced by treatment with intramuscular BoNT-A [18, 19]. However, this treatment can be efficient when the increase in resistance to passive movement is associated with a predominant neural contribution while the stretching technique would probably more effective if the resistance is predominantly elastic [20, 21].

A new instrument, the NeuroFlexor (Aggero MedTech AB, Solna, Sweden) has recently been developed to quantify the neural (spasticity) and non-neural (elasticity and viscosity) components of the resisting force produced by passive extension at the wrist. This method has been shown to be valid, reliable and sensitive to change when used to measure spasticity after stroke [22–24]. However normative data from a large cohort of healthy subjects is lacking. The primary aim of this study was to obtain normative NeuroFlexor data from healthy subjects and to describe the relation to anthropometric variables. The second aim was to use the normative data to establish NeuroFlexor cut-off values in order to explore early signs of spasticity after stroke.

## Methods

### Participants

A total of 107 healthy adult subjects (55 females and 52 males; age range 20 to 68 years, mean 44.5 years) were enrolled into a single control group. The subjects were recruited from the employees and the students of Danderyd University Hospital, Stockholm, Sweden. The participant’s demographic characteristics are shown in Table 1. The exclusion criteria were disorders of the hand (neurological or rheumatologic conditions), fractures of upper limb in the previous six months, presence of pacemaker or other stimulators and pregnancy.

**Table 1:** Demographic characteristics of the healthy subjects. Data are presented for the entire group (n= 107) and related to age (range of age 20–29, n = 17; 30–39, n= 23; 40–49, n= 27; 50–59, n= 19; 60–70, n= 21)

Variables	
Age, years	
Mean (SD)	44.49 (13.99)
Min – Max	20 – 68
Gender, *n* (%)	
Male	52 (49)
Female	55 (51)
Dominant hand, *n* (%)	
Right	94 (88)
Left	13 (12)
Height, *cm*	
Mean (SD)	173.12 (9.44)
Min – Max	151 – 193
Body weight, *kg*	
Mean (SD)	71.87 (12.85)
Min – Max	49 – 110
Hand size, *mm*	
Mean (SD)	79.7 (7.66)
Min – Max	64 – 110
Total passive ROM, *angle in degrees*	
Mean (SD)	165.37 (11.42)
Min – Max	135 – 180
Maximal grip strength, *kg*	
Mean (SD)	42.43 (11.91)
Min – Max	19.2 – 70.9
Range of age	
20 – 29	
Subjects, *n* (%)	17 (16)
Gender, *n* (%)	
Male	8 (47)
Female	9 (53)
Dominant hand, *n* (%)	
Right	15 (88)
Left	2 (12)
Height, *cm*	
Mean (SD)	171.18 (8.03)
Body weight, *kg*	
Mean (SD)	64.53 (7.87)
Hand size, *mm*	
Mean (SD)	78.12 (5.47)
Total passive ROM, *angle in degrees*	
Mean (SD)	170 (9.01)
Maximal grip strength, *kg*	
Mean (SD)	38.46 (9.22)
30 – 39	
Subjects, *n* (%)	23 (21)
Gender, *n* (%)	
Male	13 (57)
Female	10 (43)
Dominant hand, *n* (%)	
Right	17 (74)
Left	6 (26)
Height, *cm*	
Mean (SD)	175.04 (10.42)
Body weight, *kg*	
Mean (SD)	71.09 (14.55)
Hand size, *mm*	
Mean (SD)	80.22 (6.52)
Total passive ROM, *angle in degrees*	
Mean (SD)	168.7 (11.4)
Maximal grip strength, *kg*	
Mean (SD)	45.53 (12.57)
40 – 49	
Subjects, *n* (%)	27 (25)
Gender, *n (%)*	
Male	12 (44)
Female	15 (56)
Dominant hand, *n (%)*	
Right	23 (85)
Left	4 (15)
Height, *cm*	
Mean (SD)	174.26 (8.95)
Body weight, *kg*	
Mean (SD)	74.61 (14.92)
Hand size, *mm*	
Mean (SD)	80.78 (9.26)
Total passive ROM, *angle in degrees*	
Mean (SD)	162.22 (11.71)
Maximal grip strength, *kg*	
Mean (SD)	45.36 (11.73)
50 – 59	
Subjects, *n* (%)	19 (18)
Gender, *n (%)*	
Male	9 (47)
Female	10 (53)
Dominant hand, *n (%)*	
Right	19 (100)
Left	0
Height, *cm*	
Mean (SD)	175.95 (10.78)
Body weight, *kg*	
Mean (SD)	73.63 (11.9)
Hand size, *mm*	
Mean (SD)	81.26 (9.57)
Total passive ROM, *angle in degrees*	
Mean (SD)	166.84 (8.86)
Maximal grip strength, *kg*	
Mean (SD)	42.68 (11.43)
60 – 70	
Subjects, *n* (%)	21 (20)
Gender, *n (%)*	
Male	10 (48)
Female	11 (52)
Dominant hand, *n (%)*	
Right	20 (95)
Left	1 (5)
Height, *cm*	
Mean (SD)	171.29 (8,91)
Body weight, *kg*	
Mean (SD)	73.57 (10.66)
Hand size, *mm*	
Mean (SD)	77.62 (5.98)
Total passive ROM, *angle in degrees*	
Mean (SD)	160.71 (12.87)
Maximal grip strength, *kg*	
Mean (SD)	38.51 (12.7)

A sample of 39 stroke patients (13 females and 26 males; age range 33 to 69 years, mean 55.4 years), recently admitted as inpatients to the department of Rehabilitation Medicine (mean time post-stroke 2–4 weeks), was assessed with the NeuroFlexor. Clinical description of patients is presented in Table 2. Inclusion criterion was first ever stroke with clinical diagnosis of arm paresis (upper limb weakness on clinical exam). Exclusion criteria were other disorders of the hand (neurological or rheumatologic conditions) and cerebellar lesions.

**Table 2:**
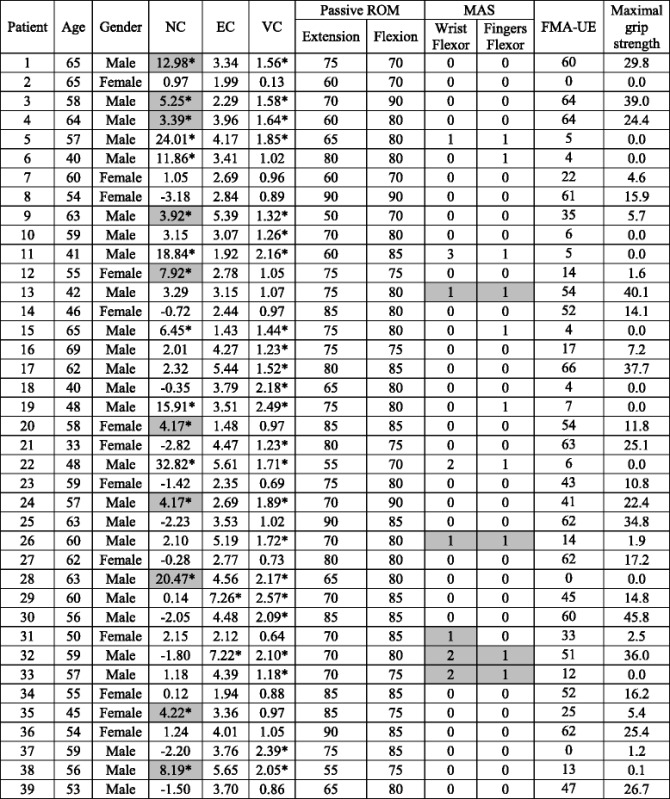
Clinical description of stroke patients

NC = neural component, EC = elastic component, VC = viscous component, Passive ROM = passive range of motion, MAS = modified Ashworth scale, FMA-UE = Fugl-Meyer assessment of the upper extremity. Pathological values of neural, elastic and viscous components in relation to cut-off values obtained by adding three standard deviations to mean are marked (*). Grey areas highlight (i) stroke patients with NC higher than cut-off value (≥3.4 *N*) without clinical detection of spasticity according to MAS and (ii) stroke patients with positive MAS scores but with NC within normal limits

Ethical approval was obtained from the Regional ethical review board in Stockholm, and written informed consent was required of all participants in accordance with the Declaration of Helsinki.

### Study design

This was a cross-sectional study with a single test session beginning with a health status questionnaire and recording of anthropometric measurements: height, body weight and hand size (approximated by the distance between wrist joint and the third metacarpal heads). Passive range of motion of the wrist was measured using a goniometer, with the subject seated with the elbow in 90° of flexion and fingers extended. Maximal grip strength was measured using the Jamar isometric dynamometer [25]. The mean value from three attempts of the dominant hand was recorded. Clinical assessment of upper limb function in stroke patients also included the modified Ashworth scale and the Fugl-Meyer assessment of the upper extremity (FMA–UE) [26].

**Fig. 1:**
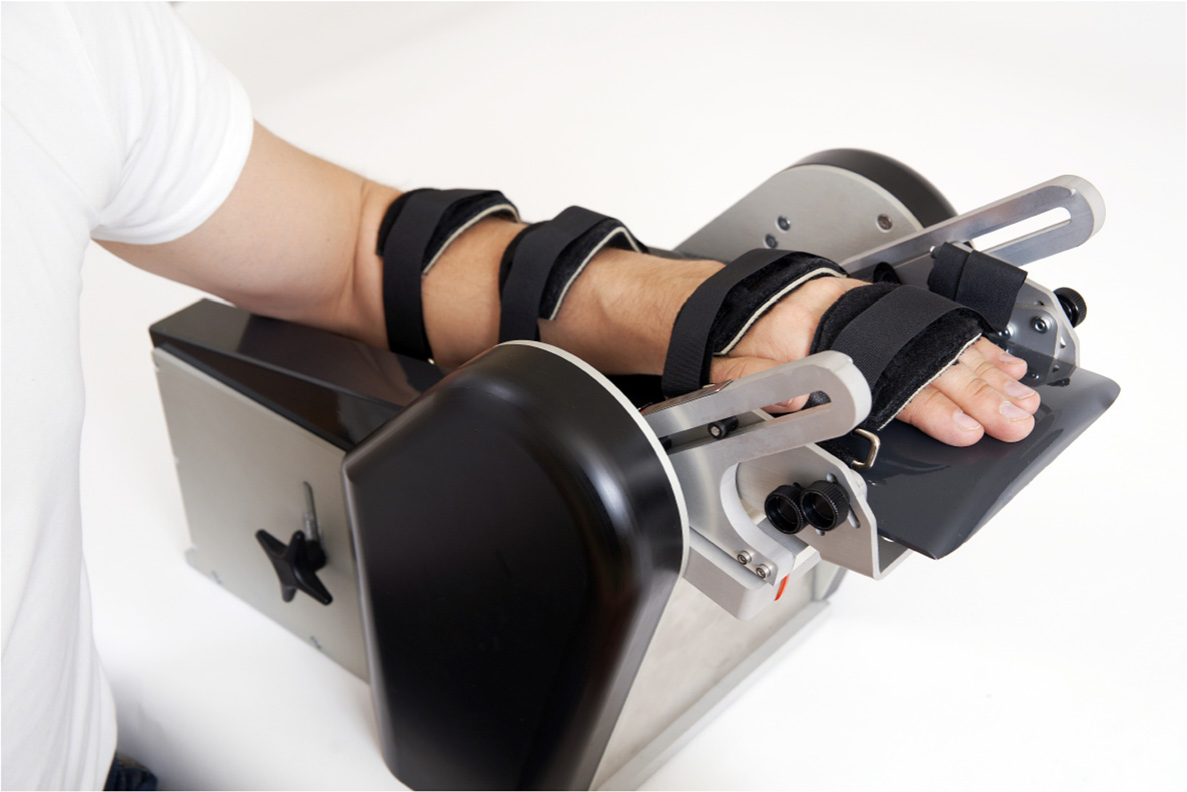
NeuroFlexor measurement device. The NeuroFlexor instrument showing the position of the hand with the metacarpophalangeal joints in slight flexion and the fingers completely extrended, and with the wrist axis of rotation aligned with the device. The instrument passively extends the wrist joint in a 50° range of motion with a starting angle of 20° of palmar flexion, and the movement is performed at controlled slow and fast velocities (5 and 236°/s, respectively)

The NeuroFlexor (www.aggeromedtech.com; Fig. 1) was used to quantify passive movement resistance during wrist extension and to calculate the contributing components. Measurements were performed on the dominant hand in healthy subjects and on the impaired hand in stroke patients.

### NeuroFlexor variables and procedures

**Fig. 2:**
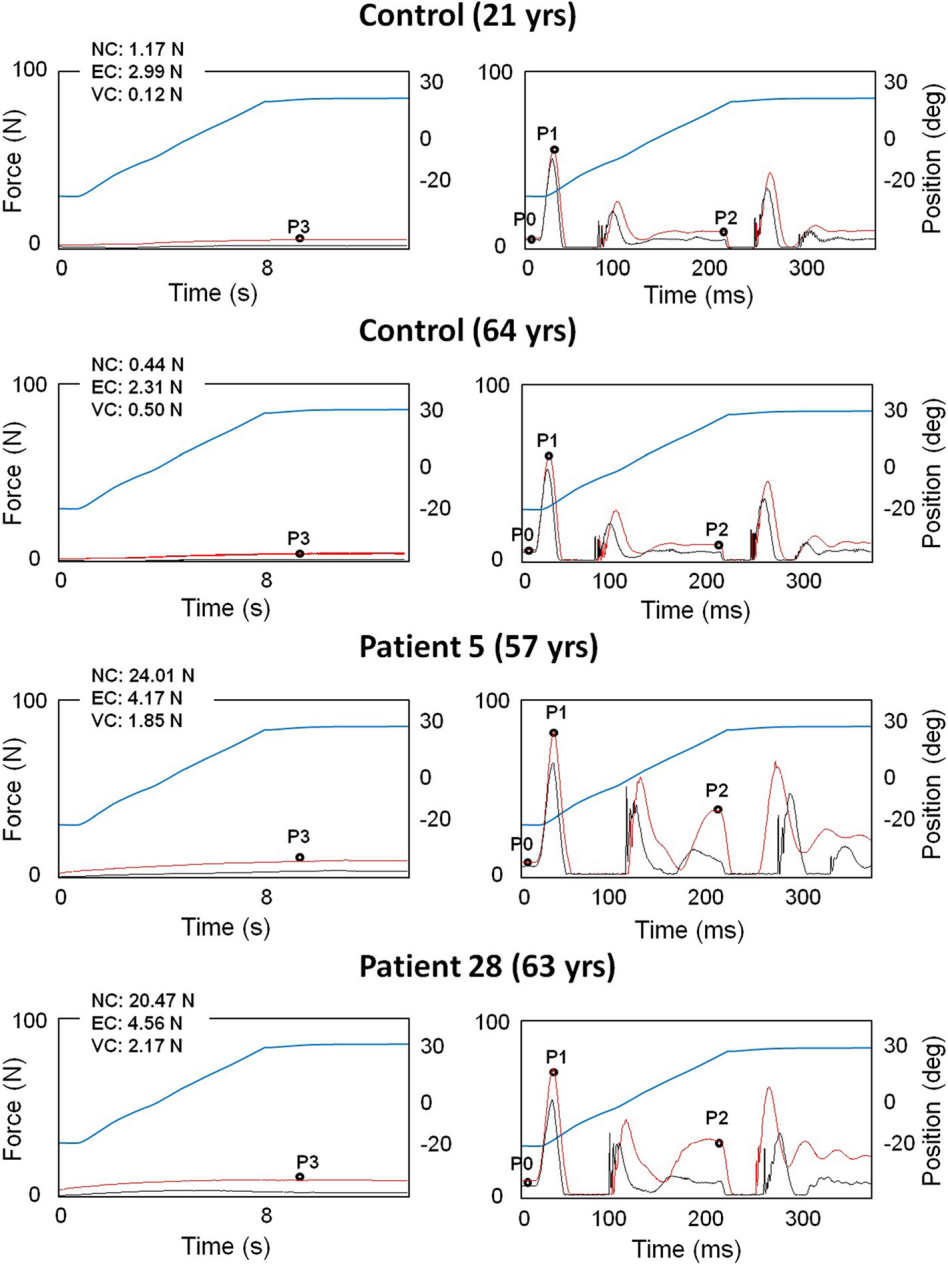
NeuroFlexor force traces. Example resistance profiles (N, newton) during slow and fast velocity movements in a young and old healthy subject and in two stroke patients. Blue traces show the angle of wrist movement (from flexion to extension). Red traces show mean resisting force from repeat trials and black traces shows mean resistance profiles when device runs without hand. Four time points are automatically identified by the software: P3 1 s after slow passive stretch; P0 in the beginning of the fast movement, P1 the first peak and P2 the peak towards the end of the fast movement. Values of neural (NC), elastic (EC) and viscous components (VC) are shown for each participant. Both healthy subjects show similar force profiles and have similar NC, EC, and VC components despite differences in age. In the patient examples the force increased during the fast movements (P2). While both patients presented NC values above normative cut-off (>3.4 N), only Patient 5 had a positive MAS score (see Table 2)

The NeuroFlexor method has been previously presented and validated in other studies on spasticity after stroke [22–24]. The biomechanical model allows separating the passive movement resistance at the wrist into active force produced by muscle contractions induced by stretch reflexes and passive mechanical components: inertia, resting tension, viscosity and elasticity (see examples in Fig. 2). The variables are briefly described below.

### Resting Tension (P0)

Resting tension reflects the tonic muscle tension of the hand before onset of stretch.

### Inertia Component (IC)

Inertia is the force resisting the acceleration of the hand and depends on the mass of the hand and the movable platform and the acceleration (*IC = m x a*, where *m* is the mass of hand and platform, and *a* is the acceleration). In the model the mass of the hand was estimated to be 0.6 % of body weight.

### Elastic Component (EC)

Elasticity is a length-dependent resisting force that increases as muscles and tendons are stretched. High EC values thus reflect a reduced elasticity of stretched tissues. In the model, EC was recorded 1 s after the end of the passive slow movement (5°/s, P3; see example in Fig. 2), thus minimizing possible contribution from stretch reflexes.

### Viscous Component (VC)

The viscosity is the force produced by friction from neighbouring tissues, for example sliding muscle fibres. The viscosity depends on the velocity of the muscle stretch and is highest during the initial acceleration and continues at a lower level during the remaining muscle stretch. In the model, the early viscosity component was defined as the resisting force that remained after the inertia component had been subtracted from the initial peak of the total resisting force at P1 (*VC*_*P1*_ *= Total force*_*P1*_*– IC*). The later viscosity had to be approximated; there is a rather stable relationship between the early and late viscosities described by Halaki et al. [27], in which the late viscosity at P2 is about 20 % of the early viscosity at P1 (*VC = (Total force*_*P1*_*– IC) x 0.2*). The late viscosity, at the end of the movement, was taken as the VC measure.

### Neural Component (NC)

The muscle stretch can activate spinal stretch reflexes with a latency of about 40 ms, followed by later stretch evoked responses adding to the first muscle contraction. In the model, the NC was estimated at the maximal extension at the end of the passive movement (P2) by subtracting the elasticity and viscosity components from the total force. (*NC = Total force*_*P2*_*– (EC + VC)*)

In this study, the NeuroFlexor measurements were performed according to the standardized procedure in previous studies [22–24] The participants were seated comfortably, with the elbow in 90° of flexion, the forearm in pronation and the dominant hand placed on the device platform. They were instructed to relax during the testing session, which consisted of passive extension of the wrist at two velocities, slow (5°/s) and fast (236°/s). The total range of wrist movement was 50°, between a starting angle of 20° of palmar flexion to 30° of extension. For each participant, one value of NC, EC and VC in Newton was calculated by dedicated software using recordings from nine fast and four slow passive movements. Resistance profiles were also obtained when the device ran empty (without hand; see resistance trace examples in Fig. 2) to enable the biomechanical model to isolate forces originating from the hand [22].

### Statistical analysis

The data were analyzed using the Statistical Package for the Social Sciences (SPSS). Descriptive statistics were shown as mean, standard deviation (SD) and frequencies (%). Cut-off scores for NC, EC, VC and resting tension were obtained by adding 3 SD to the mean [28]. For comparison, cut-off values were also calculated using prediction reference limits (99 % confidence interval, CI) obtained from linear regression of each component with age. Small negative NC values may occur due to slight differences in placement of the hand in relation to the centre of the platform force sensor [22]. The healthy population was divided by gender and into five age groups (20–29, 30–39, 40–49, 50–59, 60–70 years old) in order to obtain age and gender specific cut-off limits. Parametric methods of analysis were applied since the variables were not severely skewed (Skewness value for NC = 0.45, EC = 0.07 and VC = 0.81). Pearson’s correlation was used to test for relation between age, anthropometric data and NeuroFlexor variables (*r*). One-way ANOVA was used for studying differences related to gender. For post-hoc analysis Fisher's least significant difference (LSD) test was used.

In stroke patients, Spearman rank correlation was used to test correlations between NeuroFlexor measurements and the clinical scales scores (*r*_*s*_) since some data were not normally distributed (Shapiro-Wilk’s test, *p* < 0.05 and skewed distribution). Mann–Whitney *U* test was used to compare the NC, EC, VC and resting tension values between stroke patients and healthy subjects. The significance level was set at *p* ≤ 0.05.

## Results

### Normative data and cut-off values

NeuroFlexor recordings were similar across healthy subjects leading to small variations in components (Fig. 2). In the healthy group (*n* = 107) NC was 0.8 ± 0.9 N (mean ± SD), EC was 2.7 ± 1.1 N, VC was 0.3 ± 0.3 N and resting tension was 5.9 ± 1 N. Thus EC was found to be the component contributing the most to passive movement resistance in healthy subjects. The cut-off value (according to mean + 3SD) established for NC was 3.4 N, for EC was 6 N, for VC was 1.1 N and for the resting tension was 9 N. Less conservative prediction reference limits were obtained from 99 % CI limits of the linear regression related to age and also separately by gender, as shown in Table 3 and 4.

**Table 3:** Cut-off values for measurement with NeuroFlexor instrument obtained by adding three standard deviations to mean (N, newton)

	Mean	Std. Deviation	Min – Max	Cut-off
NC	0.80	0.87	−0.99 – 3.02	3.4
EC	2.66	1.11	−0.41 – 5.66	6
VC	0.28	0.27	−0.25 – 1.26	1.1
Resting tension	5.88	1.03	2.71 – 8.46	9

*NC* neural component; *EC* elastic component; *VC* viscous component

**Table 4:** Prediction reference limits for measurement with NeuroFlexor instrument obtained from a linear regression analysis (99 % CI) related to age and gender (N, newton)

	30 years	40 years	50 years	60 years	70 years
NC	3.0	3.0	3.1	3.2	3.3
EC					
*All population*	5.8	5.6	5.3	5.1	4.9
*Male*	6.6	6.2	5.8	5.4	5.1
*Female*	4.8	4.7	4.7	4.6	4.5
VC	0.91	1.0	1.0	1.0	1.1
Resting tension					
*All population*	8.2	8.5	8.6	8.8	9.0
*Male*	8.8	8.9	9.0	9.1	9.3
*Female*	7.6	7.9	8.1	8.4	8.7

*NC* neural component; *EC* elastic component; *VC* viscous component

### Relation to age and anthropometric data

In healthy subjects, a significant correlation was found between height and EC (*r* = 0.31, *p* = 0.01), and height and resting tension (*r* = 0.37, *p* = 0.01). Thus, taller subjects had higher EC and P0 values. Body weight also correlated positively with resting tension (*r* = 0.42, *p* = 0.01). Hand size did not correlate with any NeuroFlexor variables. Age did not correlate significantly with NC (*r* = 0.08) or with VC (*r* = 0.2) but did correlate negatively with EC (*r* = −0.3, *p* = 0.01). EC was thus lower in older compared to younger subjects. There was not found a significant correlation between age and height (*r* = 0.021). There were no gender differences for NC or VC. However, EC and resting tension were higher in males compared to females (*F* = 12, *p* = 0.001 and *F* = 12.8, *p* = 0.001, respectively). Total passive ROM (mean 165° ± 11°) did not relate to age and was similar in males and females.

### Use of cut-off values for early detection of spasticity

**Fig. 3:**
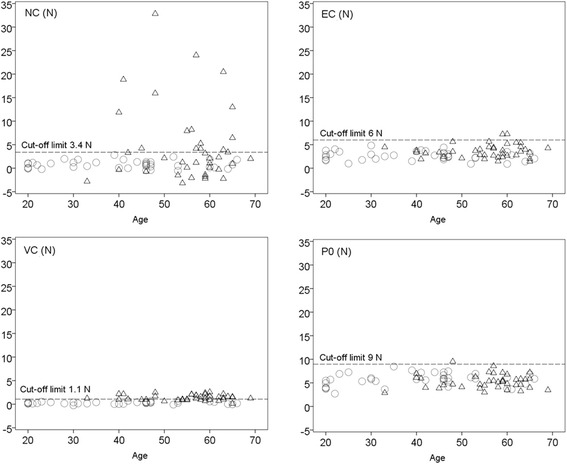
Scatter plots NeuroFlexor variables. Scatter plots of neural (NC), elastic (EC) and viscous components (VC) and resting tension (P0), (N, newton) in healthy population (circles) and stroke patients (triangles). Note the increased NC above cut-off in many stroke patients

Individual NeuroFlexor recordings showed increased resistance profiles during passive stretch of the affected hand in some stroke patients (examples shown in Fig. 2). In the stroke group (*n* = 39) NC was 4.8 ± 8.1 N (mean ± SD), EC was 3.7 ± 1.4 N, VC was 1.4 ± 0.6 N and resting tension was 5.3 ± 1.5 N. Thus NC was the component contributing the most to the passive movement resistance in stroke patients. Mann–Whitney *U* test indicated that stroke patients had higher NC, EC and VC compared to the control group (*U* = 1487.5, *p* = 0.008; *U* = 1258.5, *p* = 0.000; *U* = 128.5, *p* = 0.000, respectively). P0 was statistically significantly higher in healthy subjects than in stroke patients (*U* = 1455, *p* = 0.005). According to mean + 3SD cut-off values, some stroke patients showed pathologically high NC, EC and VC values (illustrated in Table 2 and in Fig. 3). Resting tension was above cut-off in only one patient (Patient 19), showing that both healthy subjects and patients were equally relaxed at the beginning of the stretch. Sixteen stroke patients had NC values at or above the cut-off value of 3.4 N (mean + 3SD). Two patients showed EC values above cut-off (>6 N) and 23 patients had VC values above cut-off (>1.1 N). The age and gender specific linear regression analysis for EC gave the same result, with two patients above cut-off.

### Relation to clinical measures

In the stroke group, total passive ROM (mean 153° ± 14°) was statistically significantly different from pROM values measured in the healthy subjects (*U* = 977, *p* = 0.000). Clinical MAS ratings did not correspond well with pathological NC (values > cut-off). NC was above cut-off in ten patients without signs of spasticity according to MAS (i.e., MAS = 0, Table 2). MAS scores were >0 in certain patients (*n* = 5) that had NC within normal range. For example, Patient 13, 26, 31, 32, and 33 had MAS > 0 in finger and/or wrist flexors but had NC values < 3.4 N (Table 2). Of the NeuroFlexor variables only NC correlated with upper limb function as measured with the FMA–UE (*r*_*s*_ = −0.38, *p* = 0.05). NC also correlated negatively with total passive ROM in patients (*r*_*s*_ = −0.38, *p* = 0.05). Thus patients with high NC values had lower FMA–UE scores and lower total passive ROM at the wrist.

## Discussion

The present study provided normative NeuroFlexor data from a large cohort of healthy subjects. This data allowed a detailed description of how age, gender and anthropometric measurements relate to NeuroFlexor components. Healthy population-based cut-off values proved useful for detection of pathologically high neural and non-neural components of the resisting force produced during passive wrist extension in stroke patients. These results are promising for the early clinical detection of spasticity after stroke.

### Normative data and cut-off values

The cut-off values obtained using two different methods showed some minor differences (Table 3 and 4). For the NC, the mean + 3SD approach resulted in a slightly more conservative cut-off value (3.4 N) while the linear regression cut-offs varied little across age groups. This was expected since no relation between NC and age was found. The absence of correlation with age is in accordance with previous neurophysiological studies which did not find decreased motoneuronal excitability or changes in tonic stretch reflex with aging [29–31]. VC cut-off values were also similar with the two approaches and again no effect of age was found. In contrast, small differences were found in EC and resting tension cut-offs. This was in line with the relation of these two variables to age and gender. EC/height ratio was similar between gender (0.023 for the males and 0.017 for females) and this suggests that the gender effect is likely explained by greater muscle mass in males compared to females. The reason for the reduction of EC with age was less clear, given that age is associated with increasing muscle stiffness [32–34]. However, aging also leads to reduced muscle mass and increased fat deposits [35]. A decreased proportion of muscle to adipose tissue in the forearm should lead to a reduced EC and could thus explain our findings. Indeed, our findings are in accordance with previous reports of reduced passive resistance with age [36].

In future research or clinical use, we recommend to use the slightly more conservative mean + 3SD cut-off when investigating NC and VC and the age and gender specific cut-offs when investigating EC and resting tension. This approach should limit false positives in detection of pathological values.

### Use of cut-off values for early detection of spasticity

EC contributed the most to passive movement resistance in healthy subjects while the passive resistance in stroke patients was predominantly neural in origin. This is in line with data presented in previous studies showing that NC is often increased after stroke, reflecting stretch reflex hyperexcitability [22, 37–39].

Sixteen patients (41 %) had pathologically high NC at 2–4 weeks after first-ever stroke. A positive score of MAS occurred in 11 out of 39 patients (28 %) and only 6 of these had NC above cut-off values. MAS still remains the most common clinical assessment of spasticity even if there is an increasing number of studies questioning its validity and reliability [12, 13, 40]. The limitations of the MAS likely explain the poor correspondence with the quantitative NC measures in this study. Both false positives (patients with MAS > 0 with NC < cut-off) and false negatives (patients with MAS = 0 with NC > cut-off) occurred. The joint angular velocity may affect the perceived resistance in Ashworth assessment as reported in literature [13, 41] while the speed in NF measurement is constant and high (236°/s) to be able to elicit the stretch reflex. Our findings suggest that errors in MAS ratings can be either positive or negative. It is moreover important to consider that MAS is better suited for estimation of spasticity in patients with moderate to severe muscle tone and in later phase after stroke since the MAS ratings correspond better to objective measurements in the chronic phase after stroke [22]. Other studies have also shown similar discrepancies with MAS measurements when using quantitative biomechanical approaches to measure spasticity [12]. The cut-off values also allowed detection of pathological non–neural components. EC was higher than the established cut-off in two patients and VC was above in 23 patients. Although pathologically high VC was detected in many patients the absolute values were low (Fig. 3). The VC changes may represent development of fibrosis and changes in the extracellular muscle matrix [42].

The occurrence of spasticity in this study was higher than reported in previous studies. Sommerfeld et al. [3] reported that 20 % of the patients exhibited spasticity in the upper extremity within 1 week and 18 % after 3 months; Wissel et al. [43] reported a prevalence of any spasticity of 25 % within 1 week, 27 % at 6 weeks and 22 % at 6 months; Lundström et al. [7] 17 % after 12 months and Welmer et al. [44] 19 % in the first 1–2 weeks and 20 % at 18 months after stroke. In all of the above mentioned studies, spasticity was defined as 1 point or more on the MAS. Watkins et al. [45] reported a considerably higher prevalence of any spasticity (38 %) according to both MAS and the Tone Assessment Scale, while spasticity measured using only MAS was present in 27 % of the patients. The discrepancy in the prevalence estimate between this study and the literature might be related to the age of the patients (younger in this study) as some evidence suggests that younger subjects develop more spasticity than older subjects [10, 44]. Furthermore, it is important to consider that patients in this study (inpatients in Department of Rehabilitation Medicine) probably presented more severe stroke than did the patients in other studies of unselected samples.

### Study limitations

This study had some limitations. Firstly, this study did not include a representative sample of all stroke patients but a sample of patients admitted to a department of Rehabilitation Medicine and this may explain the high prevalence of spasticity. Thus caution should be taken when comparing the observed prevalence of spasticity with prevalence data from other studies. Secondly, a small number of stroke patients was included. However, the results showed that the cut-off values from the larger healthy subject group were valuable in detecting abnormally high values in the neural and non-neural components measured with the NeuroFlexor.

## Conclusion

This study provides NeuroFlexor reference data from a healthy population and describes relationships with age, gender and anthropometric variables. The reference data allowed defining cut-off values that made it possible to detect spasticity in the early phase of recovery after stroke. The cut-off values are also promising for the detection of non-neural changes in viscosity and elasticity of stretched muscle in patients. Further studies are needed to investigate the importance of the NeuroFlexor components for the development of muscle contracture and for the sensorimotor recovery of upper limb function after stroke [46].

## Abbreviations

NC: neural component of NeuroFlexor
EC: elastic component of NeuroFlexor
VC: viscous component of NeuroFlexor
P0: resting tension of NeuroFlexor
Passive ROM: passive range of motion
MAS: modified Ashworth scale
FMA-UE: Fugl-Meyer assessment of the upper extremity

## References

[1] Lance JW, Feldman RG, Young RR, Koella WP (1980). Spasticity: disordered motor control. Symposium Synopsis.

[2] Francisco GE, McGuire JR (2012). Poststroke spasticity management. Stroke.

[3] Sommerfeld DK, Eek EU, Svensson AK, Holmqvist LW, von Arbin MH (2004). Spasticity after stroke: its occurrence and association with motor impairments and activity limitations. Stroke.

[4] Thibaut A, Chatelle C, Ziegler E, Bruno MA, Laureys S, Gosseries O (2013). Spasticity after stroke: Physiology, assessment and treatment. Brain Inj.

[5] Sommerfeld DK, Gripenstedt U, Welmer AK (2012). Spasticity after stroke: an overview of prevalence, test instruments, and treatments. Am J Phys Med Rehabil.

[6] Opheim A, Danielsson A, Alt Murphy M, Persson HC, Sunnerhagen KS (2014). Upper-limb spasticity during the first year after stroke: stroke arm longitudinal study at the University of Gothenburg. Am J Phys Med Rehabil.

[7] Lundström E, Terént A, Borg J (2008). Prevalence of disabling spasticity 1 year after first-ever stroke. Eur J Neurol.

[8] Ward AB (2012). A literature review of the pathophysiology and onset of post-stroke spasticity. Eur J Neurol.

[9] Opheim A, Danielsson A, Alt Murphy M, Persson HC, Sunnerhagen KS (2015). Early prediction of long-term upper limb spasticity after stroke: Part of the SALGOT study. Neurology.

[10] Lundström E, Smits A, Terént A, Borg J (2010). Time-course and determinants of spasticity during the first six months following first-ever stroke. J Rehabil Med.

[11] Bohannon RW, Smith MB (1987). Interrater reliability of a modified Ashworth scale of muscle spasticity. Phys Ther.

[12] Alibiglou L, Rymer WZ, Harvey RL, Mirbagheri MM (2008). The relation between Ashworth scores and neuromechanical measurements of spasticity following stroke. J Neuroeng Rehabil.

[13] Fleuren JF, Voerman GE, Erren-Wolters CV (2010). Stop using the Ashworth Scale for the assessment of spasticity. J Neurol Neurosurg Psychiatry.

[14] Abolhasani H, Ansari NN, Naghdi S, Mansouri K, Ghotbi N, Hasson S. Comparing the validity of the modified modified Ashworth scale (MMAS) and the modified Tardieu Scale (MTS) in the assessment of wrist flexor spasticity in patients with stroke: protocol for a neurophysiological study. BMJ Open. 2012;2(6). doi: 10.1136/bmjopen-2012-001394. 10.1136/bmjopen-2012-001394 PMC3532966 23166123

[15] Platz T, Vuadens P, Eickhof C, Arnold P, Van Kaick S, Heise K (2008). REPAS, a summary rating scale for resistance to passive movement: item selection, reliability and validity. Disabil Rehabil.

[16] Patrick E, Ada L (2006). The Tardieu Scale differentiates contracture from spasticity whereas the Ashworth Scale is confounded by it. Clin Rehabil.

[17] Malhotra S, Pandyan AD, Day CR, Jones PW, Hermens H (2009). Spasticity, an impairment that is poorly defined and poorly measured. Clin Rehabil.

[18] Ward AB, Wissel J, Borg J (2014). Functional goal achievement in post-stroke spasticity patients: the BOTOX® Economic Spasticity Trial (BEST). J Rehabil Med.

[19] Kwakkel G, Meskers CG (2015). Botulinum toxin A for upper limb spasticity. Lancet Neurol.

[20] Bovend'Eerdt TJ, Newman M, Barker K, Dawes H, Minelli C, Wade DT (2008). The effects of stretching in spasticity: a systematic review. Arch Phys Med Rehabil.

[21] Smania N, Picelli A, Munari D (2010). Rehabilitation procedures in the management of spasticity. Eur J Phys Rehabil Med.

[22] Lindberg PG, Gäverth J, Islam M, Fagergren A, Borg J, Forssberg H (2011). Validation of a New Biomechanical Model to Measure Muscle Tone in Spastic Muscles. Neurorehabil Neural Repair.

[23] Gäverth J, Sandgren M, Lindberg PG, Forssberg H, Eliasson AC (2013). Test-retest and inter-rater reliability of a method to measure wrist and finger spasticity. J Rehabil Med.

[24] Gäverth J, Eliasson AC, Kullander K, Borg J, Lindberg PG, Forssberg H (2014). Sensitivity of the NeuroFlexor method to measure change in spasticity after treatment with botulinum toxin A in wrist and finger muscles. J Rehabil Med.

[25] Hammer A, Lindmark B (2003). Test-retest intra-rater reliability of grip force in patients with stroke. J Rehabil Med.

[26] Fugl-Meyer AR, Jääskö L, Leyman I, Olsson S, Steglind S (1975). The post-stroke hemiplegic patient: a method for evaluation of physical performance. Scand J Rehabil Med.

[27] Halaki M, O’Dwyer N, Cathers I (2006). Systematic nonlinear relations between displacement amplitude and joint mechanics at the human wrist. J Biomech.

[28] Kuwabara S, Ogawara K, Misawa S, Mori M, Hattori T (2002). Distribution patterns of demyelination correlate with clinical profiles in chronic inflammatory demyelinating polyneuropathy. J Neurol Neurosurg Psychiatry.

[29] Chung SG, Van Rey EM, Bai Z, Rogers MW, Roth EJ, Zhang LQ (2005). Aging-related neuromuscular changes characterized by tendon reflex system properties. Arch Phys Med Rehabil.

[30] Yeo W, Ada L, O’Dwyer NJ, Neilson PD (1998). Tonic stretch reflexes in older able-bodied people. Electromyogr Clin Neurophysiol.

[31] Lin FM, Sabbahi M (1998). The aging effects on the EMG and mechanical responses of the human wrist flexor stretch reflexes. Electromyogr Clin Neurophysiol.

[32] Alnaqeeb MA, Al Zaid NS, Goldspink G (1984). Connective tissue changes and physical properties of developing and ageing skeletal muscle. J Anat.

[33] Ochala J, Frontera WR, Dorer DJ, Van Hoecke J, Krivickas LS (2007). Single Skeletal Muscle Fiber Elastic and Contractile Characteristics in Young and Older Men. J Gerontol A Biol Sci Med Sci.

[34] Narici MV, Maffulli N, Maganaris CN (2008). Ageing of human muscles and tendons. Disabil Rehabil.

[35] Rice CL, Cunningham DA, Paterson DH, Lefcoe MS (1989). Arm and leg composition determined by computed tomography in young and elderly men. Clin Physiol.

[36] Gajdosik RL, Vander Linden DW, Williams AK (1999). Influence of age on length and passive elastic stiffness characteristics of the calf muscle-tendon unit of women. Phys Ther.

[37] Ibrahim IK, Berger W, Trippel M, Dietz V (1993). Stretch-induced electromyographic activity and torque in spastic elbow muscles. Differential modulation of reflex activity in passive and active motor tasks. Brain.

[38] Powers RK, Marder-Meyer J, Rymer WZ (1988). Quantitative relations between hypertonia and stretch reflex threshold in spastic hemiparesis. Ann Neurol.

[39] Kamper DG, Harvey RL, Suresh S, Rymer WZ (2003). Relative contributions of neural mechanisms versus muscle mechanics in promoting finger extension deficits following stroke. Muscle Nerve.

[40] Burridge JH, Wood DE, Hermens HJ (2005). Theoretical and methodological considerations in the measurement of spasticity. Disabil Rehabil.

[41] Sorinola IO, White CM, Rushton DN, Newham DJ (2009). Electromyographic response to manual passive stretch of the hemiplegic wrist: accuracy, reliability, and correlation with clinical spasticity assessment and function. Neurorehabil Neural Repair.

[42] Gillies AR, Lieber RL (2011). Structure and function of the skeletal muscle extracellular matrix. Muscle Nerve.

[43] Wissel J, Schelosky LD, Scott J, Christe W, Faiss JH, Mueller J (2010). Early development of spasticity following stroke: a prospective, observational trial. J Neurol.

[44] Welmer AK, Widén Holmqvist L, Sommerfeld DK (2010). Location and severity of spasticity in the first 1–2 weeks and at 3 and 18 months after stroke. Eur J Neurol.

[45] Watkins CL, Leathley MJ, Gregson JM, Moore AP, Smith TL, Sharma AK (2002). Prevalence of spasticity post stroke. Clin Rehabil.

[46] Stinear C (2010). Prediction of recovery of motor function after stroke. Lancet Neurol.

